# Integrative Analysis of m^6^A Regulator-Mediated RNA Methylation Modification Patterns and Immune Characteristics in Lupus Nephritis

**DOI:** 10.3389/fcell.2021.724837

**Published:** 2021-09-07

**Authors:** Huanhuan Zhao, Shaokang Pan, Jiayu Duan, Fengxun Liu, Guangpu Li, Dongwei Liu, Zhangsuo Liu

**Affiliations:** ^1^Department of Nephrology, The First Affiliated Hospital of Zhengzhou University, Zhengzhou, China; ^2^Research Institute of Nephrology, Zhengzhou University, Zhengzhou, China; ^3^Research Center for Kidney Disease, Zhengzhou, China; ^4^Key Laboratory of Precision Diagnosis and Treatment for Chronic Kidney Disease in Henan Province, Zhengzhou, China; ^5^Core Unit of National Clinical Medical Research Center of Kidney Disease, Zhengzhou, China

**Keywords:** lupus nephritis, epigenetics, m^6^A RNA methylation, immune characteristics, bioinformatic analysis

## Abstract

**Background:**

There is growing evidence to demonstrate that the epigenetic regulation of immune characteristics, especially for N6-methyladenosine (m^6^A) RNA methylation. However, how m^6^A methylation is involved in lupus nephritis (LN) is still unclear. This study aimed to determine the role of m^6^A RNA methylation and their association with the immune microenvironment in LN.

**Methods:**

In total, 87 glomeruli (73 LN, 14 living healthy donors), 110 tubulointerstitium (95 LN, 15 living healthy donors), and 21 kidney whole tissue samples (14 LN, 7 controls) were included in our research to evaluate the expression levels of m^6^A regulators. CIBERSORT was used to assess the abundance of infiltrating immunocytes. The m^6^A regulator gene signature for LN was identified using LASSO-logistic regression and verified with external data. Consensus clustering algorithms were used for the unsupervised cluster analysis of m^6^A modification patterns in LN. Single-sample gene-set enrichment analysis and gene set variation analysis algorithms were employed to assess the activity of immune responses and other functional pathways. Weighted gene co-expression network analysis and protein-protein interaction networks were used to identify m^6^A methylation markers. Lastly, the Nephroseq V5 tool was used to analyze the correlation between m^6^A markers and renal function.

**Results:**

We found that the expression of m^6^A regulators was more significantly different in the glomeruli in LN compared with tubulointerstitium and whole kidney tissue. We established an m^6^A regulator signature, comprised of *METTL3*, *WTAP*, *YTHDC2*, *YTHDF1*, *FMR1*, and *FTO*, that can easily distinguish LN and healthy individuals. Two distinct m^6^A modification patterns based on 18 m^6^A regulators were determined, with significant differences in m^6^A regulator expression, immune microenvironment, biological functional pathways, and clinical characteristics. Activated NK cells, most immune responses, and HLA genes had strong correlations with m^6^A regulators. Seven m^6^A markers were identified and demonstrated a meaningful correlation with GFR, indicating that they are potential prognostic biomarkers.

**Conclusion:**

This study emphasized that m^6^A RNA methylation and the immune microenvironment are closely linked in LN. A better understanding of m^6^A modification patterns provide a basis for the development of novel therapeutic options for LN.

## Introduction

Lupus nephritis (LN) is the most common and most serious manifestation of systemic lupus erythematosus (SLE). It is also a major cause of morbidity and mortality in patients with SLE (Anders et al., 2020). Current treatments for LN are often ineffective and have strong adverse effects. In the last 50 years, only one drug has been developed for the treatment of SLE and LN, and other well-designed clinical trials have been unsuccessful (Thanou and Merrill, 2014; Narain and Furie, 2016). It is widely accepted that LN is caused by autoimmune and inflammatory responses owing to the loss of tolerance to endogenous nuclear material, which activates complement, pro-inflammatory pathways, and resident renal cells (Anders et al., 2020). Previously, the immune response to LN was mainly determined from the analysis of blood samples, which does not effectively reflect the immune state of the kidneys. Therefore, further investigation on the immune characteristics, including immune cell infiltration in LN kidney tissue, may be key in revealing its pathological mechanism and providing insight for the development of new immunotherapies for LN.

Genetic susceptibility can partially explain immune dysregulation in LN. Single-egg twins with the same gene only show a disease consistency of around 20–40%, suggesting that in addition to genetic susceptibility, epigenetics influenced by environmental factors also play an important role in SLE (Javierre et al., 2010) RNA methylation has been widely studied in epigenetic research. N6-methyladenosine (m^6^A) methylation is the most common RNA post-transcriptional modification that regulates gene expression outside of DNA sequences in eukaryotes and plays a key role in diseases progression (Meyer and Jaffrey, 2014; Huang et al., 2018). It is a reversible process mediated by an expanding list of m^6^A binding proteins (“readers”), adenosine methyltransferases (“writers”), and potential m^6^A demethylating enzymes (“erasers”) (Zaccara et al., 2019).

Current studies have demonstrated that m^6^A methylation is involved in immune regulation. For example, Han et al. (2019) discovered that the m^6^A binding protein YTHDF1 prolongs neoantigen-specific immunity through m^6^A methylation modification of mRNA. YTHDF1 is also involved in antigen cross-presentation and cross-priming of CD8^+^ T cells. Li et al. (2017) demonstrated that the m^6^A “writer” protein METTL3 regulates the homeostasis and differentiation of mouse T cells. However, no study has attempted to explore how m^6^A modification plays a role in LN, and the association between m^6^A modification and immune characteristics remains to be elucidated. The aim of this study was to clarify the role of m^6^A RNA methylation modification in LN and explore how m^6^A affects the immune status of LN.

## Materials and Methods

### Collection and Preprocessing of Data

**FIGURE 1:**
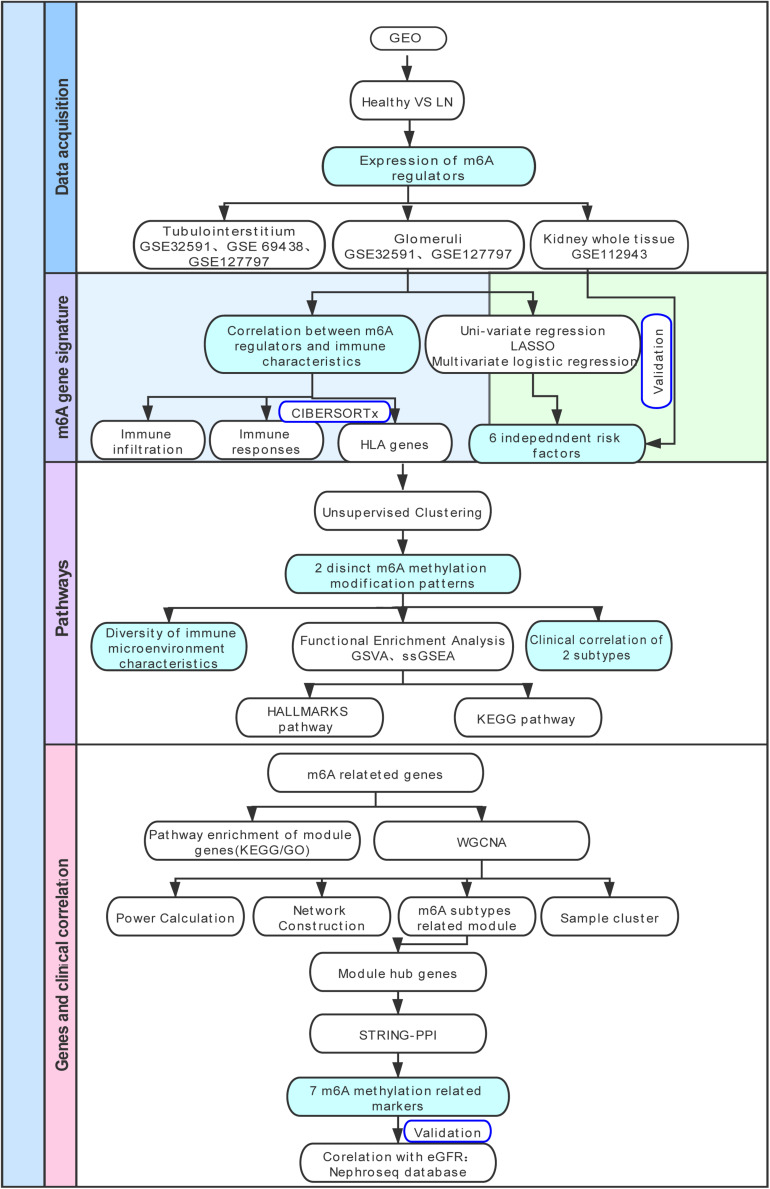
Study flow diagram. GEO, Gene Expression Omnibus; LN, lupus nephritis; HLA, human leukocyte antigen; LASSO, least absolute shrinkage and selection operator; GSVA, gene set variation analysis; ssGSEA, single sample gene set enrichment analysis; KEGG, Kyoto Encyclopedia of Genes and Genomes; GO-BP, Gene Ontology Biological Processes; WGCNA, weighted gene co-expression network analysis; GO, Gene Ontology; PPI, protein-protein interaction.

The research strategy is presented in Figure 1. We collected gene expression data of patients with LN and healthy living donors from the Gene Expression Omnibus (GEO) database.^1^ Four datasets (GSE32591 (Berthier et al., 2012), GSE69438 (Ju et al., 2015), GSE127797 (Almaani et al., 2019), and GSE112943) were selected in our study. GSE32591 contained 93 samples, which included 47 tubulointerstitium samples (32 LN samples, 15 living healthy donor samples), whereas 46 glomeruli samples (32 LN samples, 14 living healthy donor samples) were obtained from GPL14663 (Affymetrix Genechip HG-U133A). The platform for GSE69438 was GPL11670 (Affymetrix Human Genome U133 Plus 2.0 Array). It contained 42 tubulointerstitium samples, including 16 LN samples. The platform for GSE127797 was GPL24299 (Affymetrix Human Transcriptome Array 2.0), which contained 47 LN tubulointerstitium samples and 41 LN glomeruli samples. GSE127797 was the only dataset that included the pathological classifications of patients with LN. GSE112943 contained 21 whole kidney tissue samples, including 14 LN and 7 control, and sequencing was performed on GPL10558 (Illumina HumanHT-12 V4.0 expression beadchip). We then divided the samples into the glomeruli, tubulointerstitium, and whole kidney tissue for subsequent analysis. In total, 87 glomeruli samples (73 LN, 14 living healthy donors), 110 tubulointerstitium samples (95 LN, 15 living healthy donors), and 21 whole kidney tissues (14 LN, 7 controls) were included in our study to evaluate the expression levels of m^6^A regulators. All probes were converted into gene symbols, and median gene expression was used to represent the average expression level when multiple probes corresponded to the same gene symbol. We normalized the expression data from different datasets using the robust multi-array average, merged them together, and used the sva library for ComBat Batch correction to remove batch effects (Leek et al., 2012).

### m^6^A RNA Methylation Regulator Detection

The m^6^A RNA methylation regulator list we used was based on previous publications (Zhao et al., 2017; He et al., 2019; Zaccara et al., 2019). Then, the R package “limma” was applied to determine expression differences of m^6^A regulators between LN samples and healthy samples (including the glomeruli, tubulointerstitium, and kidney tissues) (Ritchie et al., 2015).

### Development and Validation of m^6^A Regulator Gene Signature for LN

Univariate logistic regression was used to preliminarily screen variables in the identified m^6^A regulator list, and LASSO regression was used to select the best predictive features while fitting a generalized linear model and avoiding overfitting (Friedman et al., 2010). m^6^A regulators with non-zero LASSO regression coefficients were included in the multivariate logistic regression (MLR) analysis. The *p*-value in the MLR was based on the Wald test, and statistical significance was set at *p* < 0.05. Forest plots were drawn using the R package “ggplot2” to visually describe the results of the logistic regression. The receiver operating characteristic curve (ROC) and the average optimism of the area under the curve (AUC) quantified the predicted probabilities of the model. The risk score for each sample was calculated as follows:


$$R\,i\,s\,k\,s\,c\,o\,r\,e=\sum_{i=1}^{n}C\,o\,e\,f_{i}*x_{i}$$

where *Coef*_*i*_ indicates the coefficients of MLR and *x_i_* is the gene expression value of each m^6^A regulator.

### Correlation Between m^6^A Regulators and Immune Characteristics

The CIBERSORTx with 1,000 permutations was used to evaluate the abundance of infiltrating immunocytes.^2^ The inclusion criterion was as follows: CIBERSORT, *p* < 0.05. We conducted single-sample gene-set enrichment analysis (ssGSEA) to assess immune response activity. We downloaded these gene sets from the ImmPort database (Bhattacharya et al., 2014).^3^ Lastly, Spearman correlation analysis was used to determine the correlation between m^6^A regulators and immune characteristics.

### Unsupervised Cluster Analysis of m^6^A Modification Patterns in LN

Based on 18 identified m^6^A regulators, unsupervised cluster analysis was performed to determine distinct m^6^A subtypes using the R package “ConsensusClusterPlus,” and the consensus clustering algorithm ran 1,000 times to guarantee the robustness of clustering (Wilkerson and Hayes, 2010). The Kruskal test was used to compare the differences in m^6^A regulator expression and immune characteristics between subtypes. Principal component analysis was performed with the R package “PCA.”

### Pathway Enrichment Analysis of the Two m^6^A Patterns

We downloaded the gene sets “h.all.v7.4.symbols” and “c2.cp.kegg.v7.4.symbols” from the MSigDB database. The gene set variation analysis (GSVA) algorithm was used to calculate the pathway activation score, which was conducted using the R package “GSVA” (Hänzelmann et al., 2013). The R package “limma” was used to compare the differences in pathway activation score between two subtypes, and a *p*-value < 0.01 was the cut-off criterion (Ritchie et al., 2015).

### Identification of m^6^A Modification Pattern Markers

m^6^A modification subtypes-related differentially expressed genes (DEGs) between two distinct m^6^A subtypes (*p* < 0.0001) were defined as m^6^A related genes. m^6^A related genes were enriched in biological processes (BP), cellular component (CC), and molecular function (MF) terms in Gene Ontology (GO) and Kyoto Encyclopedia of Genes and Genomes (KEGG) pathways and were visualized with a bubble plot. We performed enrichment analysis with the cut-off criterion of the *Q*-value at < 0.05, and it was conducted using the “clusterProfiler” package (Yu et al., 2012).

Weighted gene co-expression network analysis (WGCNA) was conducted to identify m^6^A subtype-related genes and gene modules that characterize the pathways or functions of subtypes based on gene profiles using the WGCNA R package (Langfelder and Horvath, 2008). Correlations between different modules and subgroups were analyzed using Pearson’s correlation.

We used the STRING database^4^ to construct a protein-protein interaction (PPI) network for genes from the key module of the WGCNA. Visualization was performed using Cytoscape (Szklarczyk et al., 2019).^5^

### Clinical Correlation With m^6^A Pattern Markers

The Nephroseq V5 tool^6^ was used to determine the correlation between m^6^A markers and renal function. We downloaded the expression data of markers and used “ggplot2” to replot the scatter plots.

## Results

### Landscape of m^6^A RNA Methylation Regulators in LN

**FIGURE 2:**
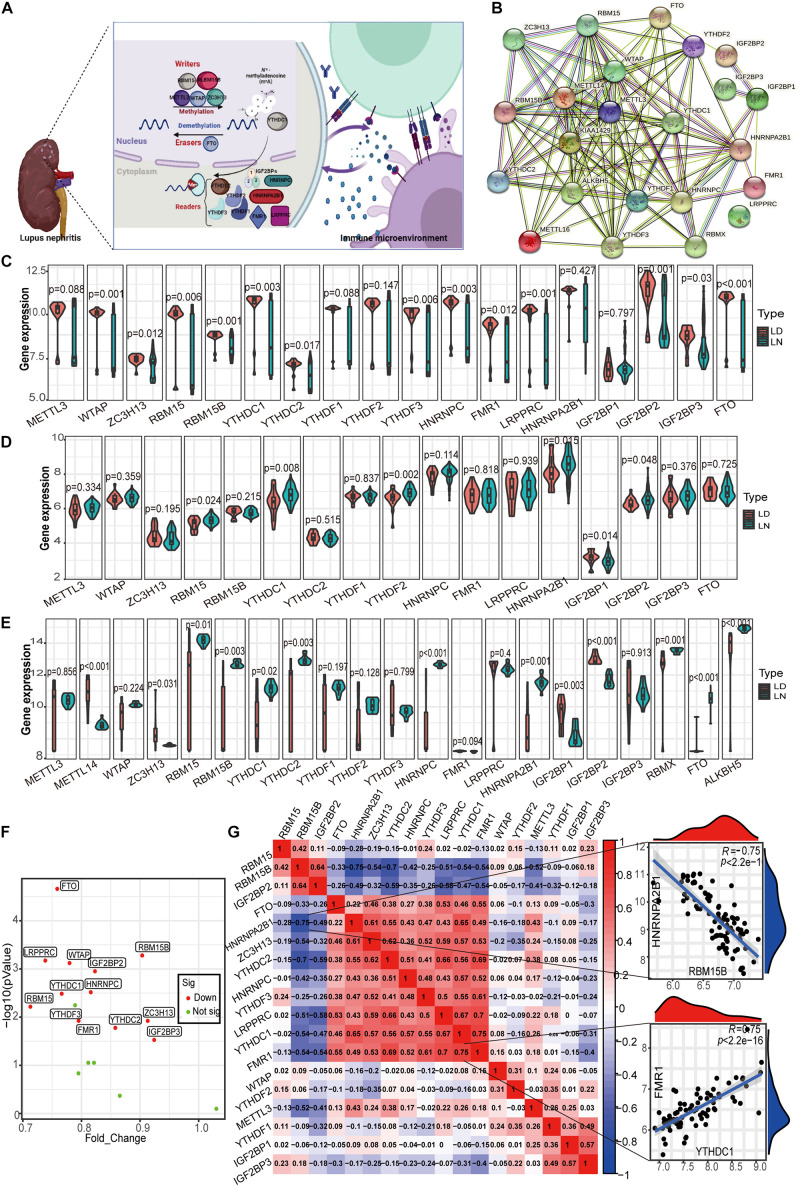
Landscape of m^6^A RNA methylation regulators in LN. **(A)** m^6^A RNA methylation modification regulated by m^6^A “writer,” “reader,” and “eraser,” which is involved in the immune microenvironment of LN. **(B)** Protein-protein interaction (PPI) network composed of 23 m^6^A regulators. **(C)** Violin plot demonstrating the expression level of 18 m^6^A regulators in glomeruli between living donor and LN. **(D)** Violin plot demonstrating the expression level of 17 m^6^A regulators in tubulointerstitium between living donor and LN. **(E)** Violin plot demonstrating the expression level of 21 m^6^A regulators in kidney whole tissue between living donor and of LN. **(F)** Volcano plot showing a summary of the expression differences of 18 m^6^A regulators between the healthy and LN patients’ glomerular samples. **(G)** Correlations between 18 m^6^A regulators in LN glomeruli samples. The two respective scatterplots show the two pairs of m^6^A regulators with the highest correlation, HNRNPA2B1 and RBM15B with the most negative correlation, and YTHDC1 and FMR1 with the most positive correlation.

Currently, there are 23 m^6^A RNA methylation regulators that have been widely studied, including 8 writers, 13 readers, and 2 erasers. Figure 2A shows the m^6^A regulators with functions and crosstalk between regulators and the immune microenvironment. The regulatory interactions of these 23 m^6^A regulators are shown in Figure 2B. First, the m^6^A regulator gene expression values of glomeruli (GSE32591 and GSE127797), tubulointerstitium (GSE32591, GSE69438, and GSE127797), and whole kidney tissues (GSE112943) of LN and healthy samples were evaluated. The expression of m^6^A regulators was the most considerably different in the glomeruli between LN and healthy samples. In total, 18 m^6^A RNA methylation regulators were identified in the glomeruli (Figure 2C). Significant expression differences in the 13 regulators (*p* < 0.05) were observed between LN and healthy samples, including WTAP, RBM15B, LRPPRC, and FTO (*p* < 0.001). Differences in the expression of m^6^A regulators between LN and healthy samples in the tubulointerstitium were not significant. As shown in Figure 2D, only six expressions altered m^6^A regulators in 17 identified m^6^A regulators. Significant expression differences in the 13 m^6^A regulators (*p* < 0.05) were observed among 21 m^6^A identified regulators in whole kidney tissue (Figure 2E). Taken together, the most significant differences in the expression of m^6^A regulators between LN and healthy samples were observed in the glomeruli. Thus, we selected the glomeruli samples for further detailed analysis.

Interestingly, the expression of all 13 altered m^6^A regulators was downregulated in LN compared with healthy samples in the glomeruli (Figure 2F). The decrease in fold change of *RBM15* was the largest among these genes, whereas the decrease in eraser protein FTO levels was the most statistically significant. Note that there was no significant difference in the expression of the well-studied writer METTL13 between LN and healthy samples. In the correlation analysis for 18 m^6^A regulators, we observed that close relationships among m^6^A regulators which means they function together (Figure 2G). It contributes to explore the specific mechanism of aberrant m^6^A modification in LN. Figure 2G also shows the two pairs of m^6^A regulators with the highest positive/negative correlation. The reader HNRNPA2B1 and writer RBM15B were the most negatively correlated, whereas the readers YTHDC1 in the nuclei and FMR1 in the cytoplasm were the most positively correlated.

### m^6^A Regulators Have the Potential to Distinguish Between Healthy and LN Samples

**FIGURE 3:**
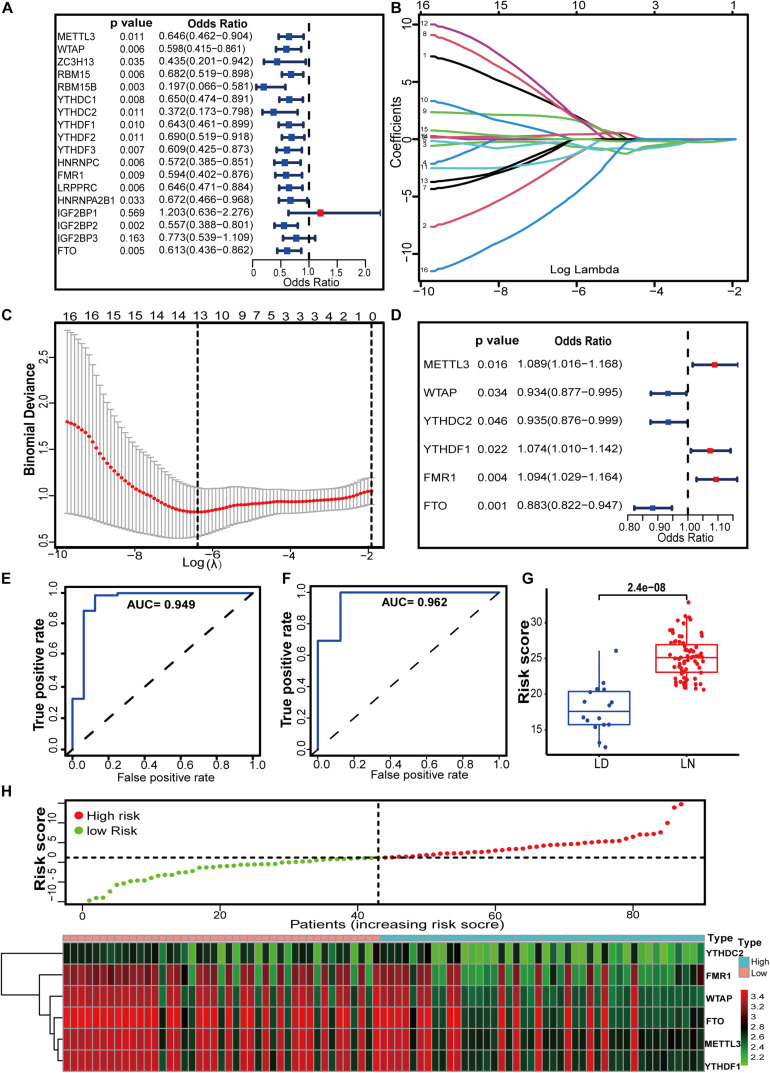
m^6^A regulators have the potential to distinguish between healthy and LN individuals. **(A)** Univariate logistic regression revealed 16 LN-related m^6^A regulators (*P* < 0.05). **(B,C)** Feature selection by LASSO regression model. **(B)** By verifying the optimal parameter (lambda) in the LASSO model, the partial likelihood deviance (binomial deviance) curve was plotted vs. log (lambda). Dotted vertical lines were drawn based on 1 SE of the minimum criteria (the 1-SE criteria). **(C)** Thirteen features with non-zero coefficients were selected by optimal lambda. A coefficient profile plot was produced against the log (lambda) sequence in **(B)**. **(D)** Multivariate logistic analysis distinguished six independent risk factors and risk scores for LN were calculated using the LASSO Logistic regression algorithm. **(E,F)** The predictive value of the m^6^A regulator gene signature in the derivation **(E)** and validation **(F)** sets by calculating the pooled AUC. 0.9 < AUC ≤ 1 indicates that the gene signature has high accuracy. **(G)** Distribution of risk scores in healthy and LN samples. **(H)** Risk score distribution based on the 6 m^6^A RNA modification regulator signature and gene expression profiles between our study groups. Patients were divided into high-risk and low-risk groups by the black dotted line, which indicates the median cut-off value.

To better understand the contribution of m^6^A regulators to the progression of LN, we established an m^6^A regulator gene signature. First, 16 regulators were selected from 18 identified m^6^A regulators by univariate logistic regression analysis (Figure 3A). LASSO regression was performed to further screen the m^6^A candidates, in which 13 regulators with non-zero coefficients were included in the multivariate logistic regression (Figures 3B,C). Finally, multivariate logistic regression analysis demonstrated that *METTL3*, *WTAP*, *YTHDC2*, *YTHDF1*, *FMR1*, and *FTO* were independently associated with LN (Figure 3D). It should be noted that the well-studied writer protein METTL3 is an independent risk factor for LN, although its expression does not differ between LN and in healthy samples. How METTL3 plays a role in LN might be an interesting topic for further exploration. For the gene signature model, the AUC for the derivation sets was 0.949, indicating that this model performed well in classifying healthy and LN samples (Figure 3E). In the independent external validation set (GSE112943), the AUC was 0.962, which suggests its ability to classify the samples (Figure 3F). In addition, Figure 3G shows that there was a significant difference in m^6^A risk scores between LN and healthy samples. The risk scores for LN were noticeably higher than those for healthy samples (Figure 3G). The distribution of risk scores and gene profiles based on the six selected m^6^A regulators is shown in Figure 3H. We observed that the expression of WTAP, YTHDC2, and FTO decreased in the high-risk group.

### m^6^A Regulators Are Related to Immune Microenvironment in LN

**FIGURE 4:**
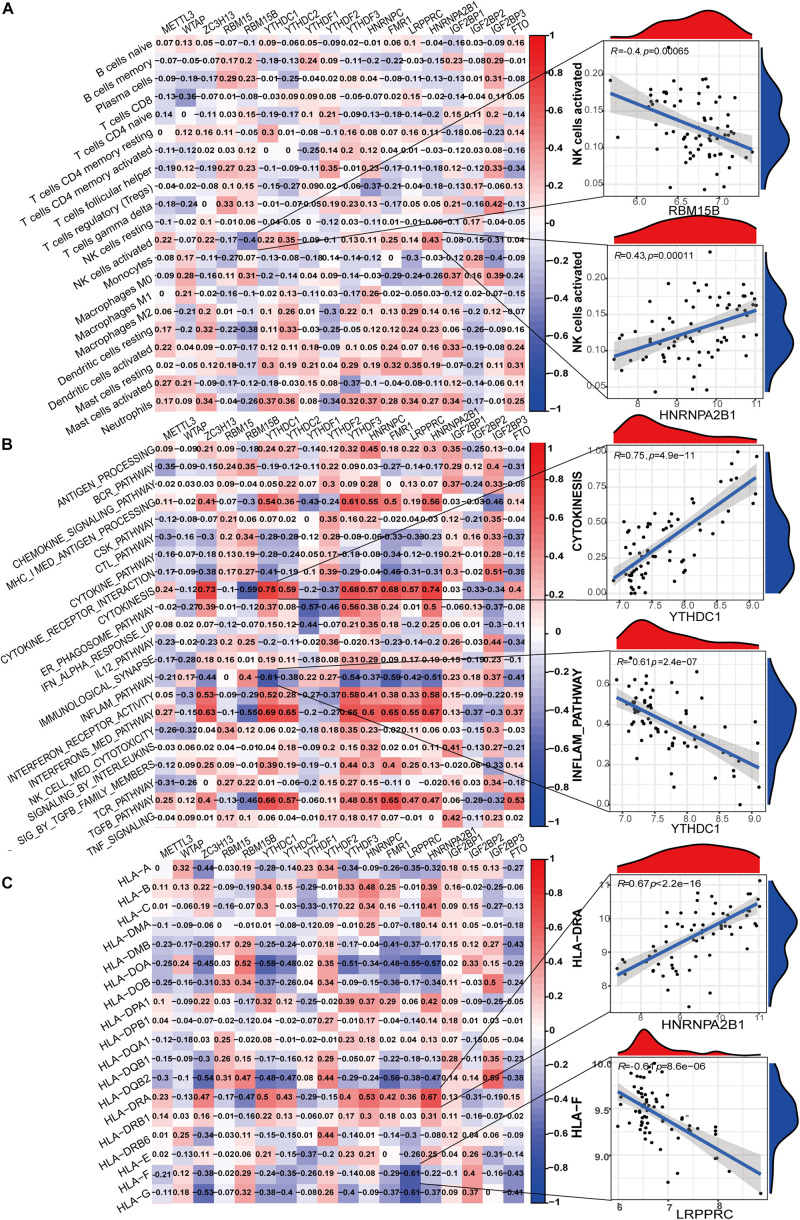
Correlation between m^6^A regulator expression and immune characteristics in LN. **(A)** Heatmap of the correlations between 18 m^6^A regulators and 21 immunocytes (eosinophils with no expression were removed in all samples). The two respective scatterplots show the m^6^A regulator and immunocyte with the highest positive or negative correlation. **(B)** Heatmap of the correlations between 18 m^6^A regulators and immune response gene sets. The two respective scatterplots show m^6^A regulators and immune response gene sets with the highest positive or negative correlation. **(C)** Heatmap of the correlations between 18 m^6^A regulators and 18 HLA genes. The two respective scatterplots show m^6^A regulators and HLA genes with the highest positive or negative correlation.

To further elucidate the relationship between m^6^A regulators and immune characteristics, we analyzed the correlations between them. Immune characteristics include immune cell infiltration, immune response activity, and Human Leukocyte Antigen (HLA) genes. The abundance of 22 infiltrating immunocytes in the glomeruli of LN was evaluated using the CIBERSORTx algorithm (Supplementary Figure 1A). Eosinophils were excluded from the correlation analysis because of the lack of expression in all samples. Several infiltrating immunocytes were correlated with m^6^A regulators but were mostly weakly correlated (Figure 4A). Among all immunocytes, activated NK cells were closely correlated with m^6^A regulators, and these were most positively correlated with HNRNPA2B1 and most negatively correlated with RBM15B. This indicates that NK-activated cell infiltration in LN is regulated by HNRNPA2B1 and RBM15B. Supplementary Figure 1B shows the expression differences of each immune response between LN and healthy samples. Correlation analysis demonstrated that most immune reaction pathways were closely related to m^6^A regulators (Figure 4B). Cytokinesis, inflammation pathway, interferon receptor activity, interferon-mediated signaling pathway, and TGF-β pathway were correlated with most of the 18 m^6^A regulators, indicating that immune dysregulation in LN is affected by m^6^A RNA methylation. Both the most positive and negative correlations between regulators and immune reactions were related to the reader protein YTHDC1, indicating that YTHDC1 exerts important functions in the cytokinesis and inflammation pathways in LN. Similarly, HLA genes were closely correlated with m^6^A regulators (Figure 4C). HLA-DRA was most positively correlated with HNRNPA2B1, with a correlation coefficient of 0.67. HLA-F was most negatively correlated with LRPPRC, with a correlation coefficient of –0.61. These indicate that HLA gene expression in LN was affected by m^6^A regulators. The differences in HLA gene expression between LN and healthy samples were shown in Supplementary Figure 1C.

### Identification of m^6^A RNA Methylation Subtypes Based on 18 m^6^A Regulators in LN and Clinical Correlation of 2 Subtypes

**FIGURE 5:**
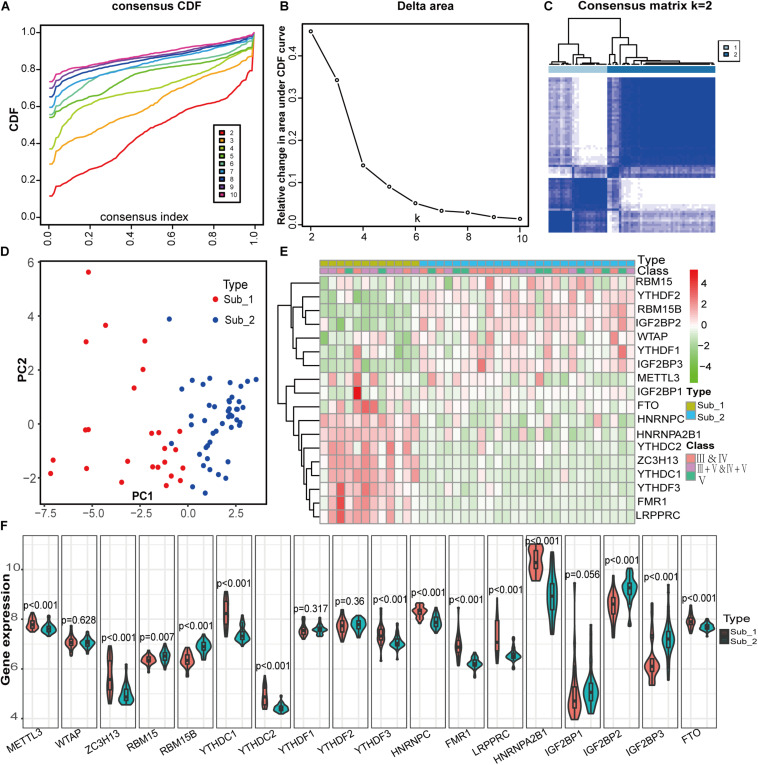
Identification of two distinct m^6^A modification subtypes in LN and clinical correlation of two subtypes. **(A)** Consensus clustering of cumulative distribution function (CDF) for *k* = 2–9. **(B)** Elbow plot shows relative change in area under CDF curve. **(C)** Consensus clustering matrix for *k* = 2. **(D)** Principal component analysis (PCA) of two m^6^A subtypes in LN. **(E)** Heatmap of the clinical features of two clusters comparing the stages of LN and gene profiles between m^6^A subtypes in GSE127797. **(F)** The two m^6^A subtypes exhibit distinct expression statuses of 18 m^6^A RNA methylation regulators.

To identify m^6^A RNA methylation modification patterns of LN, we conducted unsupervised clustering based on the expression similarity of m^6^A regulators in LN and *k* = 2 seemed to be an adequate selection resulted in 2 distinct subtypes (Figures 5A–C). Two m^6^A subtypes had significantly different populations in PCA (Figure 5D). To investigate the relationship between clinical characteristics and m^6^A subtypes, we used data from GSE127797, including the pathological stages of LN, to create a correlation heatmap. There were 38 LN samples, consisting of subtype 1 with 12 samples and subtype 2 with 26 samples. Most patients with mixed proliferative and membranous LN (class III+V and IV+V) belong to subtype 1, whereas most with pure proliferative LN (class III and IV) or pure membranous LN (class V) belong to subtype 2. Significant differences in m^6^A regulator gene profiles were observed between the two subtypes (Figures 5E,F). FTO, HNRNPC, HNRNPA2B1, YTHDC2, ZC3H13, YTHDC1, YTHDF3, FMR1, and LRPPRC were highly expressed in m^6^A subtype 1, whereas the other regulators were highly expressed in m^6^A subtype 2.

### Immune Characteristics and Biological Functional Characteristics of Two Distinct m^6^A Subtypes

**FIGURE 6:**
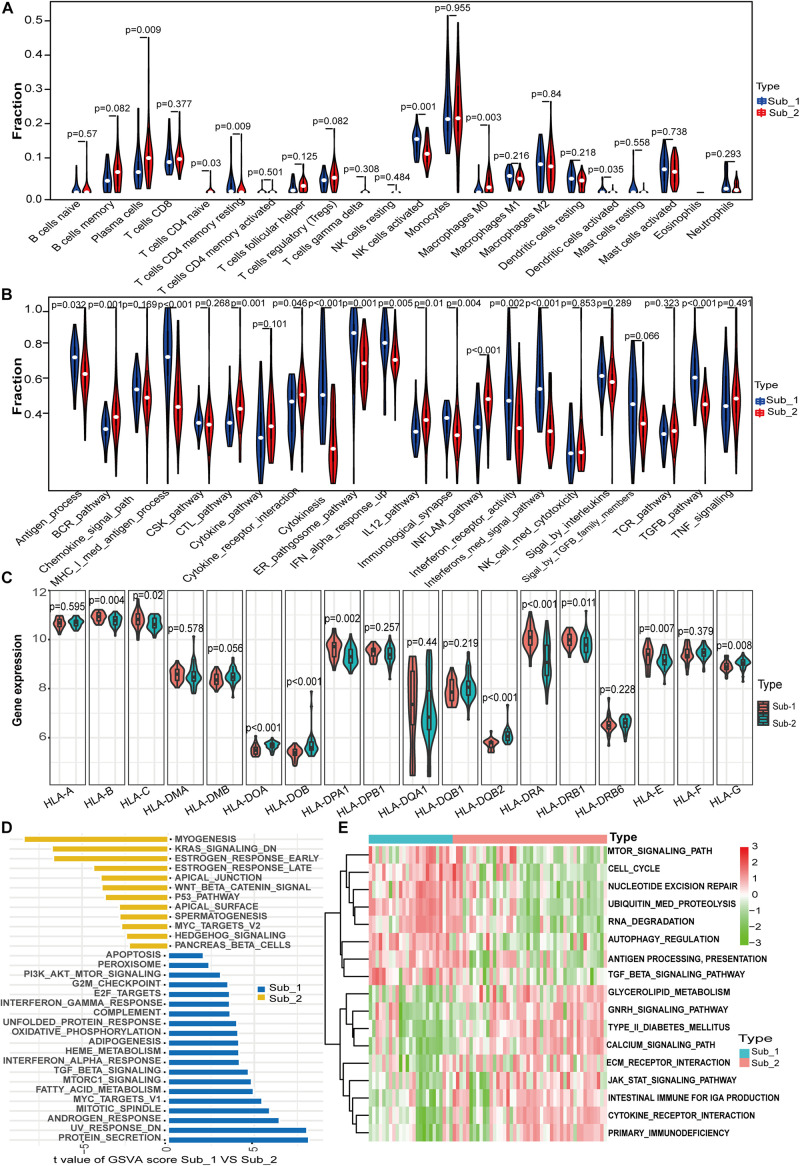
Differences in immune characteristics between m^6^A subtypes and functional enrichment analysis in two m^6^A subtypes. **(A)** Differences in abundance of 22 infiltrating immunocytes. **(B)** Differences in the activity of 22 immune response gene sets in two m^6^A subtypes. **(C)** Expression differences of 18 HLA genes in two m^6^A subtypes. **(D)** Differences in HALLMARKS pathway enrichment between m^6^A subtypes. **(E)** KEGG pathways with significant differences in enrichment between m^6^A subtypes.

To further determine the characteristics of the two m^6^A subtypes, we compared the abundance of infiltrating immune cells, activity of immune responses and HLA gene expression value between the two distinct m^6^A subtypes. More infiltrating activated NK cells (*p* = 0.001), memory resting CD4 T cells (*p* = 0.009), and activated dendritic cells (*p* = 0.03) were observed in subtype 1, whereas more plasma cells (*p* = 0.009), naïve CD4 T cells (*p* = 0.03), and macrophages M0 (*p* = 0.003) were observed in subtype 2 (Figure 6A). For immune reactions, m^6^A subtype 1 had a stronger immune response than subtype 2. There were 11 immune reactions, including MHC-I-mediated antigen processing, cytokinesis, and interferon-mediated signaling pathways, which were more active in subtype 1, whereas BCR, CTL, inflammation, and IL-12 pathways were more active in subtype 2 (Figure 6B). Different expression levels of HLA genes between the two m^6^A subtypes were also observed. For example, subtype 1 had a higher expression of HLA-C, HLA-DPA1, and HLA-DRA, whereas subtype 2 had a higher expression of HLA-DOB and HLA-DQB2 (Figure 6C). Taken together, m^6^A modification patterns were shaped with different immune characteristics, suggesting that m^6^A RNA methylation regulators might play an important role in immune microenvironment regulation in LN. To investigate the biological functional pathways that m^6^A may affect, we conducted GSVA to assess the enrichment of biological pathways. Figure 6D shows the enrichment difference of the HALLMARKS pathways between the two subtypes, indicating that protein secretion and UV-response pathways are more enriched in subtype 1, whereas myogenesis and KRAS signaling pathways are more enriched in subtype 2. Some KEGG pathways, including regulation of autophagy, TGF-β signaling pathway, and antigen processing and presentation were more enriched in subtype 1, whereas other pathways such as cytokine-cytokine receptor interaction, intestinal immune network for IgA production, and the JAK-STAT signaling pathway were more enriched in subtype 2 (Figure 6E). It should be noted that the highly enriched pathways in both subtypes both included immune-related pathways.

### Identification of m^6^A Methylation Modification Markers and Clinical Correlation of Markers With Renal Function

**FIGURE 7:**
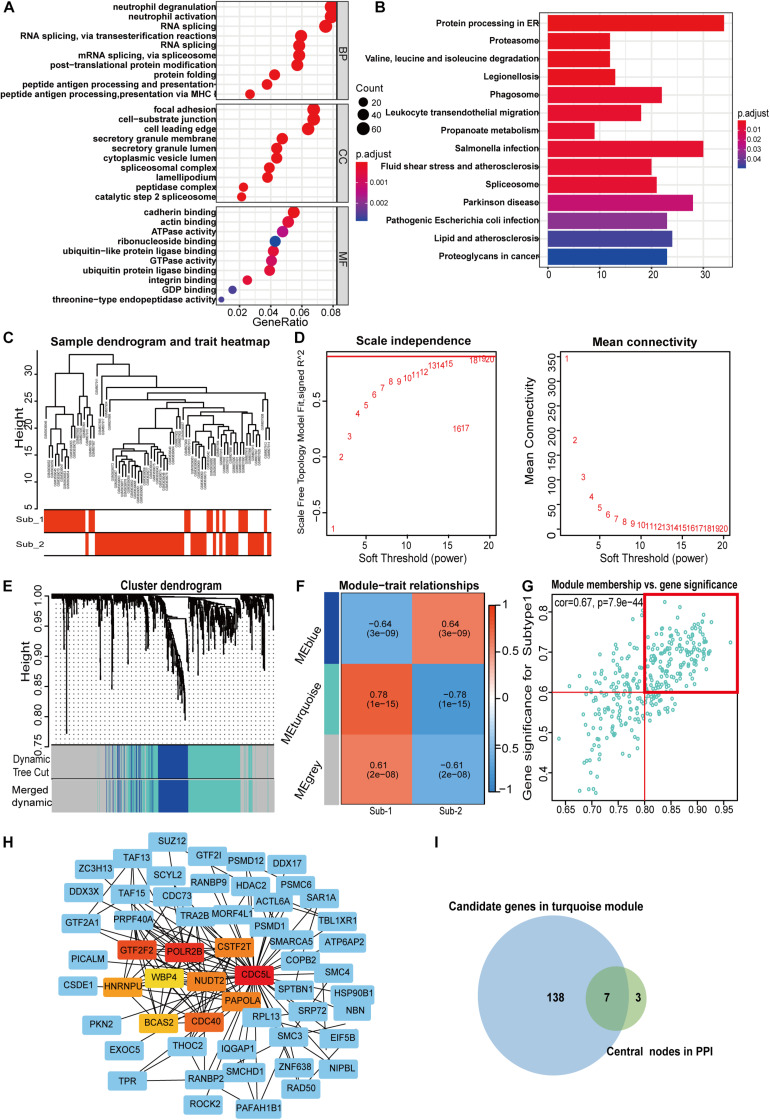
Pathway enrichment analysis of m^6^A regulator related genes **(A,B)** and identification of m^6^A methylation pattern markers in LN **(C–I)**. **(A)** Enrichment analysis of GO biological process, cellular component, and molecular function. **(B)** Bubble plot of KEGG enrichment pathways. **(C)** Clustering dendrogram of two m^6^A modification subtypes in LN. **(D)** Scale-free fitting index analysis and mean connectivity of soft threshold power from 1 to 20. **(E)** Clustering dendrograms for m^6^A regulator-related genes. According to dynamic tree cutting, the genes were clustered into different modules through hierarchical clustering and merged when the correlation of the modules is > 0.8. Each color represents each module. **(F)** Correlation heatmap between module eigen genes and m^6^A subtypes. **(G)** Scatter plot of m^6^A subtype 1 in the turquoise module. In the turquoise module, GS and MM show a very significant correlation, indicating that the genes of the turquoise module are highly related to the m^6^A modification subtype. The dots in the red box indicate that the module membership of these genes is > 0.8, and their gene significance > 0.6, meaning that these dots are the hub genes of the turquoise module. **(H)** PPI analysis network of m^6^A methylation-related genes from the turquoise module 10, the central nodes in PPI are marked in red, orange, and yellow. **(I)** Venn diagram of seven m^6^A modification markers. The central nodes of PPI (green set) were overlapped with the hub genes in the turquoise module (blue set) by weighted correlation network analysis.

**FIGURE 8:**
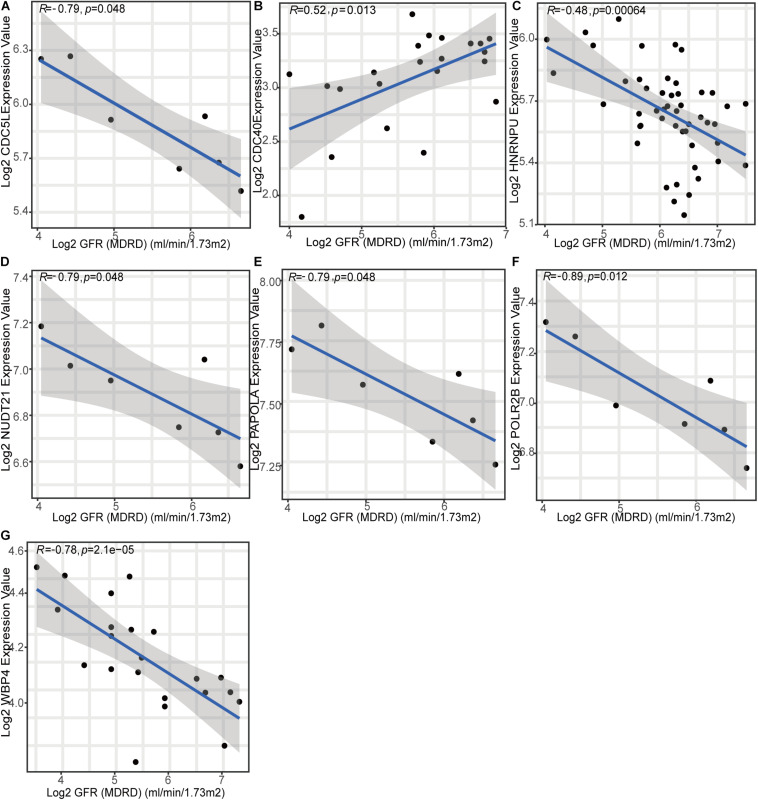
Relationship between seven m^6^A methylation pattern markers and renal function (glomerular filtration rate).

To gain further insight into which genes are involved in the biological processes affected by m^6^A regulators, we identified m^6^A-related genes, and enrichment analysis of these genes was performed. The top 10 pathways in the BP and GO were mainly RNA or protein modification pathways and immune-related pathways, such as neutrophil degranulation and MHC-I-mediated antigen processing (Figure 7A). This confirmed that m^6^A methylation modification was associated with the immune microenvironment in LN. The KEGG enrichment analysis revealed that m^6^A modification patterns-related genes were more enriched in protein processing in the endoplasmic reticulum and Salmonella infection pathways (Figure 7B). Based on m^6^A related genes, we performed WGCNA to identify module hub genes (Figures 7C–E). Three gene modules were established, including the nonsense gray module, based on their similar expression spectrum (Figure 7F). The MEturquoise module genes were most positively correlated with m^6^A subtype 1 (*R*^2^ = 0.78) (Figure 7G), indicating that MEturquoise is a key module. Then, genes in MEturquoise were used to construct the PPI network (Figure 7H). If the module membership (MM) of genes in the turquoise module was > 0.8, and their gene significance (GS) was > 0.6, these genes were considered the hub genes of the turquoise module. Finally, we overlapped the central nodes in the PPI and hub genes of the turquoise module, and seven m^6^A RNA methylation modification markers (CDC5L, CDC40, HNRNPU, NUDT21, PAPOLA, POLR2B, and WBP4) were identified (Figure 7I). To further elucidate the roles of these m^6^A markers in LN, correlation analysis between markers and GFR was carried out in the Nephroseq database (Figures 8A–G). Among the seven markers, only CDC40 was positively correlated with GFR, thus, a higher expression of CDC40 indicates better renal function in patients with LN have and may play a protective role against LN. The other six markers, CDC5L, HNRNPU, NUDT21, PAPOLA, POLR2B, and WBP4, were all negatively correlated with GFR, indicating that these genes may aggravate kidney damage in patients with LN.

## Discussion

LN is an autoimmune disease characterized by symptoms of inflammation. Immune response dysregulation mediated by genetic and environmental factors leads to the occurrence and development of LN (Anders et al., 2020). Many studies have confirmed that m^6^A methylation modification exerts critical functions in the development of diseases, especially malignancies. However, little research has been conducted on m^6^A methylation in LN. Our study is the first to investigate the roles of m^6^A regulators in LN and reveal the association with m^6^A methylation modification and immune characteristics. Firstly, significant differences in the expression of most m^6^A regulators between healthy individuals and LN were observed in the glomeruli. This is mainly because LN is a form of glomerulonephritis. We also identified an m^6^A regulator gene signature that included *METTL3*, *WTAP*, *YTHDC2*, *YTHDF1*, and *FMR1* after LASSO-logistic regression. LN and healthy samples were easily distinguished, which highlights that m^6^A methylation modification patterns differ between LN and healthy samples.

Then, we demonstrated a correlation between m^6^A regulators and immune characteristics. A series of immune reactions were considerably correlated with m^6^A regulators, especially MHC-I-mediated antigen processing, cytokinesis, inflammation pathway, and interferon-mediated signaling pathway. Most m^6^A regulators were found to be strongly correlated with the IFN signaling pathway. Recent studies have shown that type I interferon (IFN-I) is an important risk factor for the occurrence and progression of LN (Ding et al., 2021), indicating that m^6^A methylation modification plays a key role in the development of LN. Most HLA genes were closely correlated with m^6^A regulators. Studies have identified that HLA-DR3, HLA-DR4, HLA-DR11, and HLA-DR15 can promote or improve kidney damage in LN (Munroe and James, 2015; Iwamoto and Niewold, 2017). For immune cell infiltration, activated NK cells were most strongly correlated with m^6^A regulators. Activated NK cells were most positively correlated with HNRNPA2B1 and most negatively correlated with RBM15B. NK cells are an important link between the innate and adaptive immune systems. Postól et al. (2008) found that the onset of glomerulonephritis in NZBxNZW (F1) mice (SLE model) can be delayed by long-term depletion of NK cells, indicating that functional defects in NK cells may induce the development of LN (Spada et al., 2015; Segerberg et al., 2019).

However, the overall correlation between various immunocytes and m^6^A regulators was found to be generally weak. One possible reason for this is the limitations of previous technical tools. The samples collected for RNA sequencing contain very limited immune cells, which might not precisely reflect the abundance of infiltrating immunocytes (Stewart et al., 2020).

In our study, two distinct m^6^A RNA methylation modification subtypes were identified based on the m^6^A regulator gene expression profiles using unsupervised clustering. The differences between the 2 subtypes included the following aspects. As for immune characteristics, m^6^A subtype 1 of LN was characterized by increased immune response activation and a higher HLA gene expression profile. A higher abundance of infiltrating immune cells was observed in m^6^A subtype 2, including that of plasma cells, naïve CD4 T cells, M0 macrophages, and dendritic cells. Plasma cells play a key role in the development of SLE and LN (Crickx et al., 2021). A higher abundance of infiltrating T cells, memory resting CD4 T cells, and activated NK cells was found in subtype 1. The deposition of immunoglobulins produced by plasma cells in the glomeruli is the initial trigger for LN. Now, the focus of treatment for LN is targeted B-cell therapy. The two m^6^A subtypes of LN have the potential to be used to develop targeted immunotherapy.

Additionally, the pathological stages of LN in the two subtypes were also considerably different. Most patients with mixed proliferative and membranous LN (class III+V and IV+V) belong to subtype 1, whereas most with pure proliferative LN (class III and IV) or pure membranous LN (class V) belong to subtype 2. No current research has been conducted to illustrate the relationship between LN classification and immune status. Our results initially suggest that a greater activation of immune reaction pathways occurs in class III+V and IV+V, and a higher abundance of infiltrating plasma cells occurs in class III, IV, and V. In general, class III+V and IV+V have more complicated pathological changes than class III, IV, or V because their lesions are mixed proliferative lesions in class V (Parikh et al., 2020). LN classification is established according to differences in prognosis and is the gold standard for guiding treatment (Levey and Coresh, 2012; Mackay et al., 2019; Mageau et al., 2019). In our study, a strong correlation was observed between LN classification and m^6^A subtypes. As subtype1 is associated with mixed, complex, and more severe lesions compared with subtype2, uncovering the key differences between the subtypes will contribute to preventing the aggravation of LN. Molecular subtyping is a widely used strategy in malignancies, and targeted treatment plans can be formulated based on different molecular types to improve patient prognosis (Teo et al., 2019). The two m^6^A modification subtypes of LN have the potential to be considered as an alternative classification of LN. Furthermore, from a functional pathway perspective, genes of m^6^A subtype 1 are more enriched in the TGF-β signaling pathway, MTOR signaling pathway, and autophagy regulation.

Finally, seven m^6^A methylation modification markers were identified. CDC5L, HNRNPU, NUDT21, PAPOLA, POLR2B, and WBP4 were negatively correlated with GFR (an indicator of kidney function), whereas CDC40 was positively correlated with GFR. The protein encoded by CDC5L has been shown to be a positive regulator of the G2/M stage of the cell cycle. Zhou et al. (2020) found that CDC5L also regulates cell proliferation and metastasis in lung adenocarcinoma through promoter methylation. POLR2B encodes the second largest subunit of RNA polymerase II (Pol II), which is involved in RNA splicing and modification (Wang et al., 2021). CDC40, HNRNPU, WBP4, NUDT21, and PAPOLA are all involved in precursor mRNA splicing. S-adenosylmethionine (SAM) is a methyl donor for almost all cell methylation events. Scarborough et al. (2021) reported that NUDT21 regulates intracellular SAM levels. As shown, m^6^A RNA methylation modification plays a key role in mRNA splicing, suggesting that the m^6^A markers identified in this study are closely related to the m^6^A modification process. In addition, Fuyuno et al. (2016) proposed that the mutation of the WBP4 locus may lead to the occurrence of inflammatory bowel disease. The nuclear matrix protein HNRNPU is also considered as a nuclear virus dsRNA sensor for DNA and RNA viruses (Lin et al., 2010). m^6^A markers may be related to immune disorders and inflammatory responses, which again highlights that m^6^A-regulators can regulate immune characteristics. At present, research on LN mainly focuses on genetics and clinical advances, whereas epigenetic research is rare. There is also almost no research on m^6^A RNA methylation modification. We took the lead in identifying the role of m^6^A regulators in LN and exploring their relationship with immune characteristics. The various results in our study indicate that m^6^A methylation modification is a new direction for research regarding the pathogenesis of LN.

Our study has certain limitations. First, we were unable to obtain more clinical data for each patient, such as sex, age, treatment, and prognosis, for the longitudinal analysis. We could not perform a correlation analysis between m^6^A patterns, pathological stages, and other clinical characteristics of all samples. Second, we included as many samples as possible in the GEO database that met our requirements, but the sample size was still small (73 LN, 14 living healthy donors). Studies with larger sample sizes are required in the future. Additionally, the expression changes of some identified m^6^A regulators between LN and healthy samples were small and our findings were mainly obtained through bioinformatics analysis, which is needed to be further verified experimentally. However, the good predictive performance of our identified m^6^A regulator gene signature was verified using an external data set. The correlation between seven m^6^A markers identified from GEO data and kidney function was verified using data from the Nephroseq database. We have sufficient reasons to believe that m^6^A methylation plays an important role in the development of LN.

In summary, we comprehensively assessed the role of m^6^A methylation in the glomeruli of patients with LN, established an m^6^A regulators signature that can easily distinguish LN and healthy individuals, and identified two distinct m^6^A subtypes based on 18 m^6^A regulators. The two distinct m^6^A subtypes in LN were determined with significant differences in m^6^A regulators expression, immune microenvironment, biological functional pathways, and clinical characteristics. We uncovered an association between m^6^A subtypes and immune characteristics, which can be used to develop targeted immunotherapy. Moreover, seven m^6^A subtype markers were identified and all of them demonstrated a meaningful correlation with GFR, indicating that they are potential prognostic biomarkers.

## Data Availability Statement

The datasets presented in this study can be found in online repositories. The names of the repository/repositories and accession number(s) can be found in the article/Supplementary Material.

## Author Contributions

HZ, ZL, and DL conceived the project. HZ analyzed the data, explained the results, drafted the original manuscript, and drew the figures. SP and JD discussed the draft manuscript. FL and GL revised the manuscript. All the authors approved the final version of the manuscript.

## Conflict of Interest

The authors declare that the research was conducted in the absence of any commercial or financial relationships that could be construed as a potential conflict of interest.

## Publisher’s Note

All claims expressed in this article are solely those of the authors and do not necessarily represent those of their affiliated organizations, or those of the publisher, the editors and the reviewers. Any product that may be evaluated in this article, or claim that may be made by its manufacturer, is not guaranteed or endorsed by the publisher.

## Footnotes

1
http://www.ncbi.nlm.nih.gov/geo/

2
https://cibersort.stanford.edu/

3
https://string-db.org/

4
https://cytoscape.org/

5
http://v5.nephroseq.org/
