# Supplementary material for: Integrative Analysis of m6A Regulator-Mediated RNA Methylation Modification Patterns and Immune Characteristics in Lupus Nephritis
Source: Front Cell Dev Biol. 2021 Sep 7;9:724837. doi: 10.3389/fcell.2021.724837 (PMC8454410; doi:10.3389/fcell.2021.724837)
Supplement: Supplementary file 1 [file Image_1.pdf]

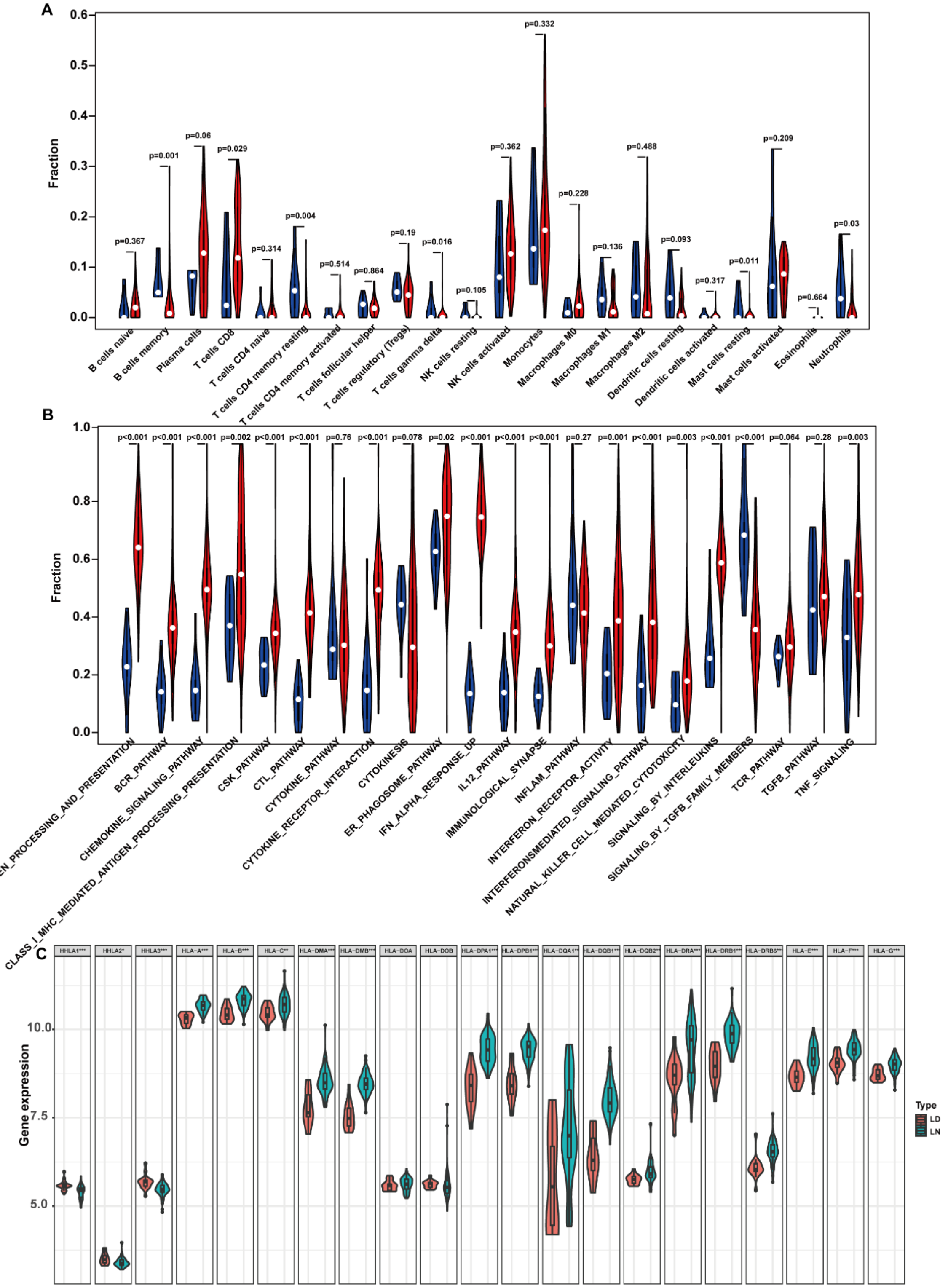

Figure S1. (A) Difference in the abundance of each immune microenvironment infiltrating cell between healthy and LN samples. (B) Difference in the activity of each immune reaction gene-set between healthy and LN samples. (C) Difference in the expression value of HLA genes between healthy and LN samples.
